# Elevated levels of salivary α- amylase activity in saliva associated with reduced odds of obesity in adult Qatari citizens: A cross-sectional study

**DOI:** 10.1371/journal.pone.0264692

**Published:** 2022-03-10

**Authors:** Neyla Al-Akl, Richard I. Thompson, Abdelilah Arredouani

**Affiliations:** 1 Qatar Biomedical Research Institute, Hamad Bin Khalifa University, Doha, Qatar; 2 College of Health and Life Sciences, Hamad Bin Khalifa University, Doha, Qatar; Medical University of Vienna, AUSTRIA

## Abstract

The relationship between salivary α-amylase activity (ssAAa) and the risk of metabolic disorders remains equivocal. We aimed to assess this relationship in adults from Qatar, where obesity and type 2 diabetes are highly prevalent. We cross-sectionally quantified ssAAa in saliva and estimated AMY1 CN from whole-genome sequencing data from 1499 participants. Linear regression was used to assess the relationship between ssAAa and adiposity and glycemic markers. Logistic regression was used to examine the association between ssAAa and occurrence of obesity or diabetes. The mean and median ssAAa were significantly lower in obese individuals. There were significant inverse associations between ssAAa and BMI, and fat mass. We detected a marked effect of ssAAa on reduced odds of obesity after adjusting for age and sex, glucose, LDL, HLD, total cholesterol, and systolic and diastolic blood pressure (OR per ssAAa unit 0.998 [95% CI 0.996–0.999], p = 0.005), with ssAAa ranging between 6.8 and 422U/mL. The obesity odds were significantly lower in the upper half of the ssAAa distributional (OR 0.58 [95% CI 0.42–0.76], p<0.001) and lower in the top versus the bottom decile of the ssAAa distribution (OR 0.46 [95% CI 0.23–0.92], p = 0.03). Our findings suggest a potential beneficial relationship between high sAAa in saliva and low odds of obesity in Qatari adults.

## Introduction

The salivary α-amylase (sAA) is a calcium metalloenzyme (EC 3.2.1.1) involved primarily in the digestion of starch in the mouth to yield a mixture of di-saccharides, tri-saccharides, and some glucose [1]. A role of sAA on starch digestion in the small intestine was also proposed, given the significant passage of sAA through the stomach due to incomplete inactivation by low pH [2]. The sAA is encoded by the *AMY1* gene, which shows extensive Copy-Number Variation (CNV) in humans, ranging from 2 to 20 copies [3–6]. Human populations show wide variability in AMY1 CNV, with those traditionally consuming low-starch diets displaying lower *AMY1* gene CNV than those consuming starchy diets [6, 7]. The *AMY1* gene CN correlates positively with both the protein levels and the enzymatic activity of the sAA in saliva [6, 8, 9], suggesting a direct effect of *AMY1* CNV on starch digestion efficiency at the oral cavity level. However, It has been suggested that the human AMY1 CNV is a minor contributor to the variation in sAA expression and activity [10]. Thus, it is not straightforward to predict an individual’s sAA activity solely from the AMY1 gene CN, and, therefore, caution must be exercised when studying the effect of AMY1 gene CN, and other genes with CNV for that matter.

Several recent nutritional studies have reported an effect of *AMY1* gene CNV on energy and glucose homeostasis [11–13]. Specifically, the importance of the role of sAA in starch digestion for postprandial glycemia was underscored. Given the release of simple sugars in the mouth following starch digestion by sAA, it was predicted that people carrying high AMY1 CN, thus high sAA levels and activity, would exhibit higher postprandial glycaemia compared to low AMY1 CN carriers. Paradoxically, however, in response to ingestion of starch, but not glucose or maltose, higher postprandial glycaemia was observed in low AMY1 CN carriers [11–13]. Mandel and her colleagues attributed this effect to an increased cephalic-phase insulin release (CPIR) in subjects with constitutively high sAA levels [11]. Of note, the importance of CPIR for postprandial glycemia was reported previously [14]. These findings may indicate that if carriers of low AMY1 CN consume food high in starch chronically, they could be at greater risk of glucose intolerance and type 2 diabetes [11, 12]. Interestingly, using metabolomics profiling, we recently showed that normoglycemic and non-obese women carrying low AMY1 CN shift their energy production to lipids [15], probably because of some degree of insulin resistance as suggested by high levels of 2-hydroxybutyrate, an early marker of insulin resistance [16].

On the other hand, an inverse association between *AMY1* gene CN and obesity was reported in many studies [5, 12, 17–27]. Additionally, basal sAA activity was inversely associated with behavioral preference for foods high in sugar [28], potentially explaining the predisposition to common obesity of low AMY1 CN carriers. It is noteworthy, however, that few studies have reported no association [29–31], or a positive association between AMY1 CN and BMI [32]. These discrepancies were attributed mainly to methodical differences regarding the correct quantification of AMY1 CN. Specifically, the appropriateness and accuracy of the qRT-PCR to measure AMY gene CN has been questioned [17, 30]. In a cross-sectional study, we recently reported that high plasma sAAa, but not high AMY1 CN, was associated with a low obesity rate in Qatari adults [4]. We have also found that high plasma sAAa was associated with reduced diabetes risk in Qatari women [33]. The prevalence of obesity and diabetes in Qatar, and the Middle East region in general, are amongst the highest in the world, with 78% of the adult population being overweight or obese and approximately 15% having type 2 diabetes [34–36]. Therefore, understanding the mechanisms that underlie these disorders and identifying markers to predict their occurrence is of paramount clinical importance.

The present cross-sectional study aimed to investigate the association between sAA activity in saliva and the odds of obesity or diabetes in a cohort of 1499 Qatari adults.

## Materials and methods

### Study participants

This cross-sectional study used baseline clinical, anthropometric, and demographic data of 1499 participants enrolled by the Qatar Biobank (QBB) between 2012 and 2018. The QBB is a research cohort and enrolls adults (age >18 years) from the general population who are either Qatari citizens or long-term residents (having lived in Qatar for at least 15 years) [36, 37]. This study included only Qatari citizens who had fasted for at least 6 hours before biospecimen collection. We did not include any pregnant women. The total number of Qatari adults is estimated to be around 155.000. Therefore, our sample represents about 1% of the Qatari adults. All the QBB cohort participants signed an informed written consent to allow the use of their data and biospecimens in research activities. The institutional review boards of QBB (IRB number: Ex-2017- RES-ACC -0054-0018) and QBRI (IRB number: 2017–001) have approved the present study.

### Anthropometric and clinical measures

For all participants, unstimulated whole saliva is collected by passive drool and immediately centrifuged and stored at -80°C until use. Measurements of the blood biochemistry are performed at the central laboratories at the Hamad Medical Corporation in Doha. To define diabetes, we used the recommendations of the American Diabetes Association; a person has diabetes if the HbA_1c_% is ≥ 6.5% (≥48 mmol/l) or the fasting plasma glucose level (FPG) is ≥126mg/dl (≥7mmom/l). Using these criteria, 224 (14.9%) and 206 (13.7%) subjects had diabetes based respectively on HbA_1c_ and FPG, with no significant difference between them (p = 0.41). We only used HbA_1c_ for our further analysis. We calculated HbA_1c_ in mmol/mol from HbA_1c_% using the formula: HbA1c(mmol/mol) = (HbA_1c_%*10.93)-23.5. Body Mass Index (BMI) was calculated as weight in kilograms divided by height in meters squared (Kg/m^2^). BMI was categorized based on White ethnic cutoff values (normal-weight: 18≤ BMI<25; overweight: 25≤ BMI<30; obese BMI≥30Kg/m^2^). For the assessment of insulin resistance, we used the Homeostasis Model Assessment of insulin resistance (HOMA-IR) equation; HOMA-IR = (fasting insulin (μU/L) x fasting glucose (mmol/L))/22.5. For the assessment of the pancreatic β-cell function, we used the Homeostasis Model Assessment β (HOMA-β) equation; HOMA-β = ((20* fasting insulin (μU/L))/(fasting glucose (mmol/L)-3.5))*100. Visceral adipose tissue and fat mass measures were obtained from iDXA data.

### Quantification of the salivary α-amylase activity in saliva

We quantified the sAA activity in saliva samples (ssAAa) using a colorimetric kinetic enzyme assay from Salimetrics (5–1902 5PK, Salimetrics, State College, PA; https://salimetrics.com/) according to the manufacturer’s instructions. Briefly, before the assay, the saliva samples are thawed to room temperature and centrifuged to remove solid particles from suspension. The enzymatic substrate in the kit is the 2- chloro-p-nitrophenol linked with maltotriose. The sAA catalyzes the conversion of this substrate to yellow-colored 2-chloro-p-nitrophenol, which absorbs 405 nm light, and the sAA enzyme activity is proportional to the increase in absorbance over time. Absorbance was measured at 405nm at 37°C in a plate reader at 1 min and then again at 3 min following substrate addition. The difference between the 1 and 3 min absorbances was calculated and multiplied by 328 to yield enzyme activity in U/mL, based on the manufacturer’s formula, which accounts for the light path of the provided wells, the millimolar absorptivity of 2-chloro-p-nitrophenol, and the dilution. The reported sAA enzyme activity values are the average of duplicate measurements.

### Estimation of AMY1 CNVs

We estimated the AMY1 gene copy number from whole-genome sequencing data, which we obtained from the Qatar genome program (QGP), a program under the QBB. The QGP has sequenced the whole genome of thousands of Qatari adults for research purposes. The data can be obtained upon request and IRB approval. We have used version 0.4 of CNVnator, which uses read-depth (RD) genome sequencing analysis for CNV discovery and genotyping [38]. Briefly, out of the WGS bam files, Chr1-aligned reads were extracted and indexed using Bamtools [39]. Read mappings were collected, and the RDs were determined using CNVnator for regions of 1 kb. The reads were partitioned into 100 base regions that were not overlapping. Each read bin was normalised by dividing the average Read Depth (RD) over all the bins by the average RD of all bins with the same GC content and multiplying by the RD of the bin. Analysis was only carried out for chr1, so normalisation was carried out over chr1 as opposed to the whole genome. Aligned reads were filtered based on their coordinates such that only those reads which were uniquely aligned to the known positions of AMY1 genes were kept. This was carried out at the final stage, using the chr1 data throughout processing. CNV counts were then calculated from the uniform read counts and filtered for regions of interest. Test CNV counts were then calculated by summing the counts for the regions of interest unique to AMY1A. We rounded the copy number of AMY1 to the nearest integer.

### Statistical analysis

We carried all the statistical analyzes using Stata 15.1/IC software (http://www.stata.com). Descriptive statistics were used to present the mean and standard deviation, median, and proportion data. The comparison of continuous characteristics between groups was performed with an unpaired t-test for independent samples. For proportions, we used the Chi^2^ test. Variables with outliers were winsorized using winsor2 command in Stata. We assessed the associations of the ssAAa or AMY1 CN with the obesity or diabetes markers with adjusted linear regression and the association of ssAAa or AMY1 CN with the odds of obesity or diabetes with adjusted logistic regression. None of the variables had more than 7% missing values and were imputed using multiple imputations by chained equations in Stata. Statistical significance was considered at p<0.05.

## Results

**Fig 1:**
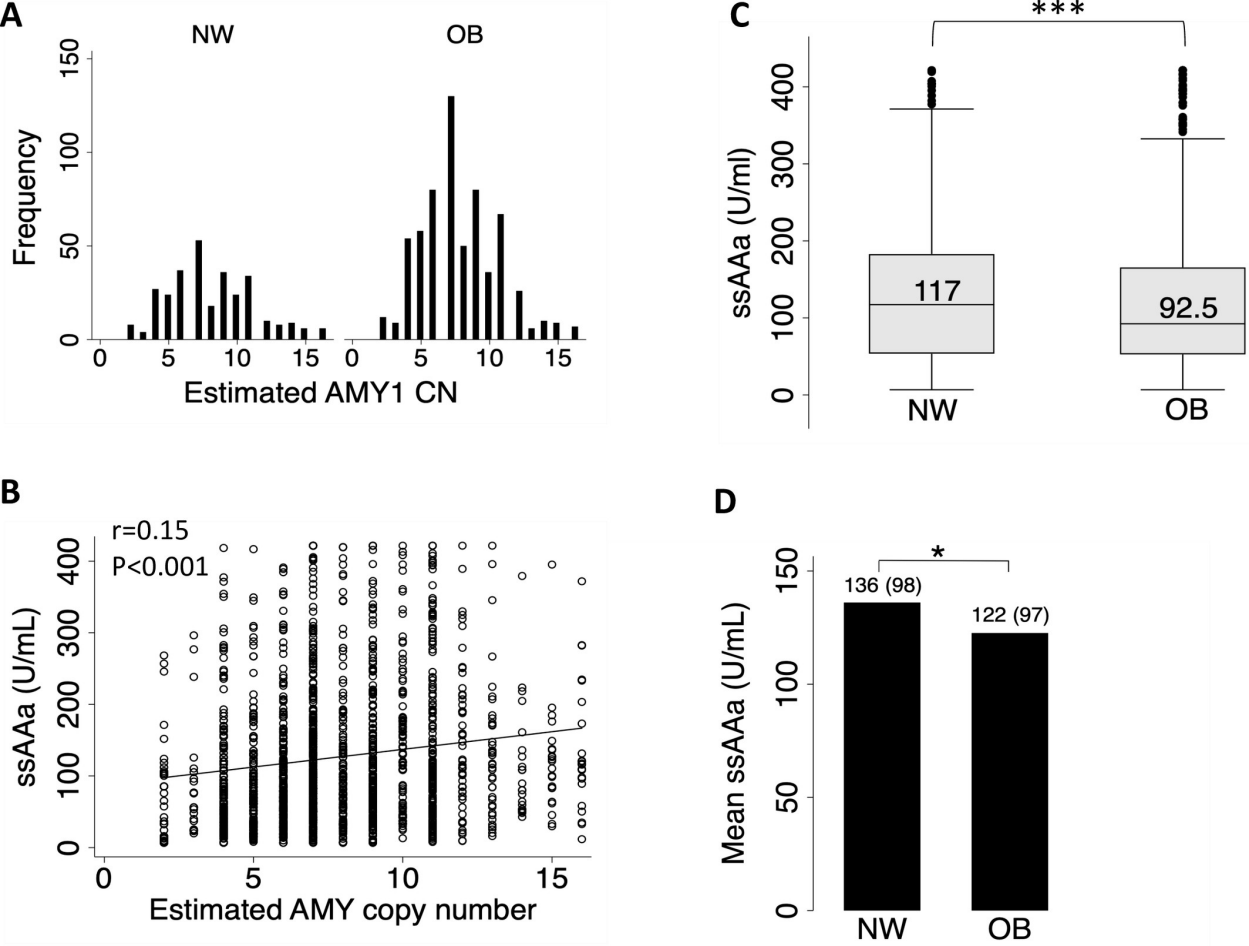
Distribution and correlation of ssAAa and AMY1. (A) Distribution of AMY1 gene CN in normal weight and obese subjects. (B) Box and whiskers plot of ssAAa in obese and normal weight subjects. (C) Correlation between ssAAa and AMY1 gene CN in total sample population. (D) Mean of ssAAa in obese and normal weight subjects. statistical significance: *** p<0.001; ** p<0.01; *0.05.

Table 1 displays the baseline features of the participants. Of the 1499 subjects surveyed (59% females), 304 (20%), 561 (37%), and 634 (42%) individuals were normal weight (NW), overweight (OW), and obese (OB), respectively. Diabetes and prediabetes affected 224 (14%) and 264 (17%) participants, respectively. The estimated AMY 1 CN ranged from 2 to 20 (Fig 1A), with no difference in mean and median AMY1 CN between NW versus OW or OB (Table 1) and between diabetes versus normoglycemic participants (7.65 ±0.19 versus 7.95 ± 0.09, p = 0.16). The ssAAa levels in our population ranged from 6.8 to 422 U/ml, with obese people having significantly lower mean and median ssAAa levels (Table 1 and Fig 1C and 1D). No such significant difference was observed based on diabetes status (The mean and median ssAAa levels were respectively 126 99U/ml and 100.4 U/ml in healthy participants, and 131.698 U/ml and 108.6 U/ml in diabetics).

**Table 1:** Baseline characteristic of the study participants.

Characteristics	Normal weight	Obese	P value
n (male/female)	304 (127/177)	634 (227/407)***	<0.001
Age (years)	37.1 (12.5)	42.6 (12.6)***	<0.001
AMY1 CN (mean)	8.1 (3.2)	7.8 (2.8)	0.1182
AMY1 CN (median)	7	7	0.279
ssAAa U/ml (mean)	136.1 (98.2)	122.6 (96.6)*	0.0468
ssAAa U/ml (median)	117	92.5***	0.001
BMI (Kg/m^2^)	22.2 (2.1)	35.0 (4.1)***	<0.001
Waist (cm)	82.9 (14.7)	93.3 (14.4)***	<0.001
Hip (cm)	102.9 (11.3)	111.1 (11.3)***	<0.001
WHR	0.8 (0.1)	0.8 (0.1)***	<0.001
FPG (mmol/l)	5.5 (1.7)	6.2 (2.5)***	<0.001
HbA_1c_%	5.7 (1.0)	6.0 (1.3)***	<0.001
Insulin (mmol/l)	9.4 (6.5)	12.9 (9.5)***	<0.001
HOMA-IR	2.4 (2.5)	3.8 (4.3)***	<0.001
HOMA-B	107.8 (63.6)	124.6 (77.7)**	0.0011
HDL (mmol/l)	1.5 (0.4)	1.4 (0.4)***	<0.001
LDL (mmol/l)	3.0 (0.8)	3.0 (0.8)	0.1367
Total cholesterol (mmol/l)	4.9 (0.9)	5.0 (0.9)	0.3015
TGs (mmol/l)	1.2 (0.6)	1.3 (0.7)***	<0.001
Bodyweight (kg)	71.5 (16.9)	84.9 (17.2)***	<0.001
Body fat (%)	27.5 (7.9)	43.6 (7.4)***	<0.001
Fat mass (KG)	16.6 (5.4)	39.8 (9.3)***	<0.001
VAT (Kg)	0.9 (0.7)	2.4 (1.6)***	<0.001

Data are mean (SD), median or proportion. AMY1 CN: AMY1 gene copy number; ssAAa: saliva salivary a-amylase; BMI: body mass, index; WHR: waist-to-Hip ratio; FPG: fasting plasma glucose; HbA_1c_: glycated hemoglobin; HOMAR-IR: Homeostatic Model Assessment of Insulin Resistance; HOMA-B: Homeostatic Model Assessment of beta cell function; HDL: high density lipoprotein; LDL: low density lipoprotein; TGs: triglycerides; VAT: Visceral Adipose tissue. The stars indicated statistically significant difference compared to normal weight.

* p<0.05,

** p <0.01,

*** p<0.001.

Pearson correlation analysis revealed a significant, positive, but weak correlation between ssAAa and AMY1 CN in our sample population (Fig 1B). We used linear regression adjusted for age and sex to assess this association further and found that each additional AMY1 gene copy increased the ssAAa by 4.8 U/ml (95% CI 3.15–6.45; p<0.001), and explained only 5.3% of the activity.

We then used the linear regression analysis adjusted for age and sex to investigate the association between ssAAa and adiposity markers (Table 2). We found that ssAAa was negatively and significantly associated with BMI (p<001) and fat mass (p<0.001). However, no significant association was detected between ssAAa and glycemic or lipid markers after adjusting for age, sex, and BMI (Table 2). We also assessed the association between AMY1 CN and adiposity markers and lipid or glycemic markers and could not detect any significant association (S1 Table).

**Table 2:** Linear regression adjusted for age and sex or for age, sex and BMI (variables in shaded cells) examining associations between ssAAa and different adiposity and glycemic markers.

Dependent variables	β (95% CI)	P value
BMI (Kg/m^2^)	-0.009 (-0.012, -0.006)***	<0.001
Waist (cm)	-0.006 (-0.01, 0.0003)	0.063
Hip (cm)	-0.001 (-0.007, 0.004)	0.583
WHR	-0.0004 (-0.00009, -0.000002)*	0.059
Fat Mass (kg)	-0.01 (-0.016, 0.004)***	<0.001
Body fat (%)	-0.001 (-0.005, 0.003)	0.560
VAT (Kg)	0.0001 (-0.0005, 0.008)	0.677
BW (Kg)	-0.007 (-0.16, -0.001)	0.106
**FPG (mmol/l)**	0.0002 (-.0008, 0.001)	0.653
**HbA1c%**	-0.00005 (-0.0007, 0.0005)	0.859
**HOMA-IR**	0.0014 (-0.0004, 0.003)	0.138
**Total cholesterol (mmol/l)**	0.0003 (-0.0002, 0.0008)	0.236
**HDL (mmol/l)**	-0.00003 (0.0002, 0.0001)	0.715
**LDL (mmol/l)**	0.0002 (-0.0002, 0.0006)	0.316
**Triglycerides (mmol/l)**	-0.00003 (-0.0003, 0.003)	0.860

The ssAAa in our cohort ranges from 6.8 to 422U/mL.

*, p<0.5;

***p<0.001.

Unadjusted logistic regression (model 1 in Table 3) revealed a significant association between ssAAa and reduced odds of obesity (OR per ssAAa unit 0.998 (95% CI 0.997–0.999, p = 0.047), with ssAAa ranging from 6.8 to 422 U/mL. This association remained significant after adjustment for age and sex (model 2 in Table 3, p = 0.003), and after controlling for glucose, HDL, LDL, total cholesterol, triglycerides, and systolic and diastolic blood pressure (Model 3 in Table 3, p = 0.005). However, we did not find any significant association between AMY1 CN and odds of obesity or between ssAAa or AMY1 CN and the odds of diabetes in our cohort (S2 Table).

**Table 3:** Logistic regression analysis examining the association between ssAAa and odds of obesity.

	OR (95% CI)	p
**Model 1**	0.998 (0.997–0.999)	**0.047**
**Model 2**	0.997 (0.996–0.999)	**0.003**
**Model 3**	0.998 (0.996–0.999)	**0.005**

Model 1: unadjusted. Model 2: adjusted for age and sex. Model 3: Model 2 adjusted for glucose, HDL, LDL, total cholesterol, triglycerides, systolic and diastolic blood pressure.

Given that the mean ssAAa is significantly lower in obese individuals, we compared the odds of obesity between the upper and lower half of ssAAa distribution based on the median ssAAa (98.4U/ml). Using unadjusted logistic regression (model 1 in Table 4), we found that the odds of obesity is significantly lower in the upper half as compared to the lower half (used as reference (OR = 1)) of ssAAa distribution (OR 0.66, 95% CI 0.50–0.87; p = 0.003). The effect of ssAAa on odds of obesity remained significant after adjustment for age and sex (model 2 in Table 3), and for glucose, HDL, LDL, total cholesterol, triglycerides, systolic and diastolic blood pressure (Model 3 in Table 3). We further run a logistic regression adjusted for age and sex (Model 2 in Table 5) using the top and bottom (reference) deciles of the ssAAa distribution and found that the odds of being obese in the top decile is 0.45 times the odds of being obese in the bottom decile (OR 0.45; 95% CI 0.22, 0.86; p = 0.017). The OR remained significant (OR 0.46; 95% CI 0.23, 0.92, p = 0.03) even after we further adjusted for glucose, HDL, LDL, total cholesterol, triglyceride, systolic and diastolic blood pressure (Model 3 in Table 5). These results suggest a beneficial effect of high ssAAa on odds of obesity in our cohort.

**Table 4:** Logistic regression analysis examining the association between ssAAa and odds of obesity between individuals with low and high ssAAa.

	OR (95% CI)		P values
	Low ssAAa group (ssAAa<98U/mL)	High ssAAa group (ssAAa≥98U/mL)	
**Model 1**	1 (reference)	0.66 (0.50–0.87)	0.003
**Model 2**	1 (reference)	0.58 (0.43–0.77)	<0.0001
**Model 3**	1 (reference)	0.58 (0.42–0.76)	<0.001

Model 1: unadjusted. Model 2: adjusted for age and sex. Model 3: model 2 adjusted for glucose, HDL, LDL, total cholesterol, triglycerides, systolic and diastolic blood pressure.

The median ssAAa is 98, which is used to created low and high ssAAa groups.

**Table 5:** Logistic regression analysis adjusted for age and sex examining the association between ssAAa and odds of obesity in top and bottom deciles of the ssAAa distribution.

	OR (95% CI)		*P values*
	Bottom decile	Top decile	
**Model 1**	1	0.58 (0.30–1.11)	*0*.*04*
**Model 2**	1	0.45 (0.22–0.86)	*0*.*017*
**Model 3**	1	0.46 (0.23 0.92)	*0*.*030*

Model 1: unadjusted. Model 2: adjusted for age and sex. Model 3: model 2 adjusted for glucose, HDL, LDL, total cholesterol, triglyceride, systolic and diastolic blood pressure. The ssAAa ranges from 6.8 to 422 u/mL.

## Discussion

In the present cross-sectional investigation, we report that high salivary α-amylase activity (ssAAa) in the saliva is significantly associated with reduced odds of obesity in Qatari adults. However, there was no significant association between the ssAAa and the odds of diabetes nor between AMY1 CN and odds obesity or diabetes.

Although the primary function of the ssAA is in the early digestion of starch in the oral cavity, its activity might affect the postprandial glycemic response independently of starch digestion/absorption in the small intestine, but possibly through an effect on the cephalic phase of insulin secretion [9]. Furthermore, the ssAAa may impact processes related to the perception of texture and taste of food, satiety, and oral health [3, 9]. Indeed, basal ssAAa was shown to be inversely associated with behavioral preference for foods high in sugar [28], which might explain the predisposition to weight gain and obesity of low amylase individuals [5]. We previously showed that individuals with low AMY1 gene CN have an increased β-oxidation of fatty acids and reduced cellular glucose uptake [15], indicating that the sAA may have other functions extending beyond its role in digestion. It is worth noting that the expression of AMY1 is not restricted to salivary glands and that a high AMY1 expression was reported in adipose tissue [5]. Furthermore, variation in the activity of ssAAa might influence gut microbiota through dietary carbohydrate processing [40, 41]. Lately, low AMY1 gene CN was associated with abundance of the metabolically favorable gut *Prevotella* in Mexican children and adults [40, 42]. Also, an association was reported between plasma sAA activity and lactate, a product of complex intestinal carbohydrate fermentation [17]. Further investigations are required to fully understand how the ssAAa might contribute to the modulation of the function of the gastrointestinal tract in humans, and directly or indirectly affect food absorption or appetite.

Our results revealed a weak, positive, but significant correlation between ssAAa and AMY1 CN, suggesting that the human AMY1 gene CN is not the only factor that contributes to the variation in sAA expression. This findings corroborate similar findings reported earlier by Carpenter and his colleagues in a cohort from UK [10]. Contrary to what has been reported by others in other population [5, 18–21, 23–27], we failed to see any association between AMY1 gene CN and the obesity or glycemic markers in the present study. Thus, these results indicate that prudence must be exercised when studying the effect of the AMY1 gene CN, and other genes with CNV in this regard, as knowing an individual’s AMY1 gene CN is not sufficient to predict how much sAA is produced and how much of it is active. Indeed, the activity of the sAA is the factor that reflects best its current capacity and determines the rate of the degradation of polysaccharides like starch. Therefore, for the investigation of the relationship between ssAAa and the odds of metabolic disorders, the ssAAa is the most suitable parameter.

No significant associations between ssAAa the odds of diabetes were found in our regression analysis. Previously, individuals with low ssAAa were shown to exhibit higher postprandial blood glucose concentrations after starch ingestion [11, 12], and it was suggested that these individuals might be at greater risk for insulin resistance and diabetes if chronically ingesting starch-rich diets. However, Alberti and his colleagues failed to find evidence for a significant role of ssAAa in glycemic response after starch ingestion [8]. It is difficult to draw any firm conclusion from these studies, considering the limited number of participants (10,14, and10) and the various ethnicities involved (Chile (South Americans), Malaysia (South Asians), USA (mixed ethnicities)).

With respect to obesity, our findings indicate that high ssAAa was significantly associated with reduced odds of obesity (Table 3). This association seems to be driven mostly by the contribution of higher ssAAa levels, suggesting a protective effect of high ssAAa against obesity in the Qatari adults. The Qatari population, and the Middle East in general, has a diet rich in starch. Therefore, individuals with low ssAAa might be more predisposed to obesity if they consume starch chronically.

The main limitation of our study is its cross-sectional nature. Consequently, we can only report associations and the cause-effect relationship is unclear. We also did not consider other factors such as smoking status, physical activity, medications, and dietary food intake. On the other hand, the present study’s main strength is a large number of participants from a Middle Eastern population (n = 1499), compared to a recent study from Saudi Arabia (n = 200). Moreover, our data is obtained from a research biobank, and the participants are well phenotyped.

In summary, we found that high levels of ssAAa are associated with reduced odds of obesity in Qatari adults. These findings suggest a putative benefit of high production of ssAA on glucose and energy metabolism in the Qatari population where starch, in the form of rice, is a staple food. Furthermore, given the shared genetic background and dietary habits of the neighboring countries, the results of our study might be extended to those populations. Finally, to better understand the mechanisms that underlie the link between ssAAa and obesity and the cause-effect relationship, large-scale prospective studies are needed.

## Supporting information

S1 Table Linear regression adjusted for age and sex or for age, sex, and BMI (variables in shaded cells) examining associations between AMY1 CN and different adiposity and glycemic markers. (DOCX)

S2 Table Logistic regression analysis examining the association between AMY1 CN and odds of diabetes. (DOCX)
