# Supplementary material for: Elevated levels of salivary α- amylase activity in saliva associated with reduced odds of obesity in adult Qatari citizens: A cross-sectional study
Source: PLoS One. 2022 Mar 10;17(3):e0264692. doi: 10.1371/journal.pone.0264692 (PMC8912263; doi:10.1371/journal.pone.0264692)
Supplement: S1 Table — (DOCX) [file pone.0264692.s001.docx]

| **Supplementary material**  Elevated levels of salivary α- amylase activity in saliva associated with reduced odds of obesity in adult Qatari citizens: a cross-sectional study  **Neyla Al-Akl^1^, Richard I. Thompson^1^, Abdelilah Arredouani^,1,2*^**  **RESULTS**  **S1 Table. Linear regression adjusted for age and sex or for age, sex, and BMI (variables in shaded cells) examining associations between AMY1 CN and different adiposity and glycemic markers.** | | |
| --- | --- | --- |
| **Dependent variables** | **b (95% CI)** | **P value** |
| BMI (Kg/m^2^) | -.092 (-0.19, 0.005) | 0.065 |
| Waist (cm) | -0.043 (-0.28, 0.19) | 0.720 |
| Hip (cm) | -0.048 (-0.24, 0.14) | 0.620 |
| WHR | -3.34x10^-6^ (-0.001, 0.001) | 0.997 |
| Fat Mass (kg) | -0.19 (-0.388, 0.066) | 0.073 |
| Body fat (%) | -0.144 (-0.276, 0.043) | 0.525 |
| VAT (Kg) | -0.008 (-0.0, 0.013) | 0.461 |
| BW (Kg) | -0.048 (-0.338, 0.242) | 0.745 |
| FPG (mmol/l) | -0.010 (-0.046, 0.025) | 0.55 |
| HbA1c% | -0.011 (-0.031, 0.007) | 0.238 |
| HOMA-IR | -0.0001 (-0.061, 0.061) | 0.995 |
| Total cholesterol (mmol/l) | 0.001 (-0.015, 0.017) | 0.896 |
| HDL (mmol/l) | -0.0002 (-0.005, 0.006) | 0.937 |
| LDL (mmol/l) | 0.003 (-0.011, 0.018) | 0.671 |
| Triglycerides (mmol/l) | 0.0011 (-0.01, 0.012) | 0.836 |
| The ssAAa in our cohort ranges from 6.8 to 422U/mL. *, p<0.5; ***p<0.001 | | |
