# Supplementary material for: Elevated levels of salivary α- amylase activity in saliva associated with reduced odds of obesity in adult Qatari citizens: A cross-sectional study
Source: PLoS One. 2022 Mar 10;17(3):e0264692. doi: 10.1371/journal.pone.0264692 (PMC8912263; doi:10.1371/journal.pone.0264692)
Supplement: S2 Table — (DOCX) [file pone.0264692.s002.docx]

**Supplementary material**

Elevated levels of salivary α- amylase activity in saliva associated with reduced odds of obesity in adult Qatari citizens: a cross-sectional study

**Neyla Al-Akl^1^, Richard I. Thompson^1^, Abdelilah Arredouani^,1,2*^**

**RESULTS**

| **S2 Table. Logistic regression analysis examining the association between AMY1 CN and odds of diabetes.** | | |
| --- | --- | --- |
|  | **OR (95 % CI)** | **P value** |
| **Model 1** | 0.965 (0.918-1.014) | **0.165** |
| **Model 2** | 0.966 (0.917-1.017) | **0.195** |
| **Model 3** | 0.963 (0.909-1.02) | **0.201** |
| Model 1: unadjusted. Model 2: adjusted for age and sex. Model 3: Model 2 adjusted for, BMI, HDL, LDL, total cholesterol, triglyceride, systolic and diastolic blood pressure. | | |
